# ResilienCity: Resilience and Psychotic-Like Experiences 10 Years After L’Aquila Earthquake

**DOI:** 10.3389/fpsyt.2020.00077

**Published:** 2020-02-28

**Authors:** Rodolfo Rossi, Valentina Socci, Eleonora Gregori, Dalila Talevi, Alberto Collazzoni, Francesca Pacitti, Paolo Stratta, Alessandro Rossi, Giorgio Di Lorenzo

**Affiliations:** ^1^ Department of Systems Medicine, University of Rome Tor Vergata, Rome, Italy; ^2^ Department of Applied Clinical Sciences and Biotechnology, University of L’Aquila, L’Aquila, Italy; ^3^ Department of Mental Health, ASL1 Abruzzo, L’Aquila, Italy; ^4^ IRCCS Fondazione Santa Lucia, Rome, Italy

**Keywords:** psychotic-like experiences, trauma, adverse life experiences, natural disaster, earthquake

## Abstract

An earthquake hit the city of L’Aquila in central Italy in 2009, leaving the city completely destroyed and 309 casualties. Unexpectedly, lower rates of psychotic experiences in persons affected by the earthquake compared to non-affected persons were found 10 months after the earthquake. The very long-term impact of a natural disaster on the prevalence of psychotic experiences deserves more in-depth detailing. The Authors examined resilience and psychotic experiences in a university student sample of 494. No effect of direct exposure to the earthquake (odds ratio = 0.64, 95%CI [0.37, 1.11]), material damages (odds ratio = 0.86, 95%CI [0.60, 1.23]), psychological suffering (odds ratio = 1.06, 95% CI [0.83, 1.36]), or global impact severity (odds ratio = 0.92, 95%CI [0.76, 1.12]) on psychotic experiences was detected. Resilience levels did not differ between affected and non-affected persons. Resilience showed a strong protective effect on psychotic experiences (odds ratio=0.38, 95% CI [0.28, 0.51]. The protective effect of the RSA factor “Perception of Self” was significantly stronger in individuals affected by the earthquake compared to non-affected subjects. Being affected by an earthquake is not a risk factor for psychotic experiences in a university student sample, as no direct effect of the earthquake was detected after 10 years after the event. Resilience is confirmed as a strong protective factor for psychotic experiences irrespectively of large collective traumatic events. Extension of these results to a general population sample could provide interesting insights into recovery from natural disasters.

## Introduction

An earthquake (EQ) of 6.3 magnitude hit the city of L'Aquila in central Italy on April 6^th^ 2009, leaving the city completely destroyed. Three hundred and nine lost their lives and thousands were injured. After the earthquake, the entire population of the city (>60.000) was displaced either in nearby touristic areas or in tent camps within the city. Only a fraction of the population could return to their homes in the short-term, while the rest of the population had to wait several years for the reconstruction to take place. As of today, reconstruction still struggles to take place, with at least 5,000 people who are still living in transitioning housing, and the productive capacity and supporting infrastructure is still left behind, in favor of a slow, building-by-building reconstruction. The slow reconstruction procedures, more than the earthquake itself, have profoundly affected the social context of the town and its dwellers (1).

Traumatic events represent a risk factor for a plethora of psychological and psychiatric disorders, including psychotic-like experiences (PLEs). However, the equivalence of different types of trauma, such as natural disasters or interpersonal traumatic events, as risk factors for psychotic symptoms is still debated (2).

PLEs are considered a major risk factor for subsequent transition to psychotic disorders (3) as they share a number of risk factors with major psychotic disorders, including neurocognitive abnormalities (4) and traumatic events (5).

The putative mechanisms through which trauma sensitizes vulnerable individuals within the psychosis continuum include cognitive mechanisms (6), as well as stress-induced neurobiological and HPA-axis modifications (7). Interpersonal stressful life events have been repeatedly associated with PLEs (5, 8, 9). On the other hand, natural disasters affect the incidence of several mental disorders (10); however, their relevance as risk factors for PLEs is controversial. Natural disasters showed a weak effect on PLEs rate compared to interpersonal traumatic events in a large international study (2). Local studies have found opposite results (11, 12). The very long-term impact of a natural disaster on the prevalence of PLE has not been addressed so far.

We reported on the prevalence of PLEs in a group of adolescents 10 months after the 2009 EQ. Unexpectedly, lower rates of PLEs in persons affected by the EQ, compared to non-affected persons were found (13), with marginal associations between post-traumatic symptoms and PLEs (14).

A number of putative protective factors may have contributed to the evidence of lower PLEs rates in individuals affected by the EQ, including resilience. Resilience has been shown to play a strong protective effect along the entire psychosis spectrum, from PLEs (15, 16) to schizophrenia (17–19). Regarding the 2009 EQ, resilience could have buffered or moderated the stressful effects, resulting in lower levels of PLEs regardless emotional distress associated with post-traumatic symptoms.

As we previously observed, the population in L'Aquila could have expressed unexpected levels of resiliency (20, 21). Therefore, if these levels of resilience were preserved in the population, PLEs rates should not differ between affected and non-affected individuals 10 years after the event. Our goal is exploring any difference in resilience levels between affected and non-affected by the 2009 EQ. Furthermore, we aim at measuring the effects of EQ 10 years after the event on rates of PLEs in a sample of University students using an on-line survey. Our hypothesis, based on previous reports on levels of resilience in the population of L'Aquila, is that the impact of the earthquake on PLEs is negligible.

## Methods and Materials

### Sample

Participants were university students at the University of L'Aquila. Data were collected over the period of June 2018 until April 2019 using a research website designed for this purpose (LimeSurvey^®^). Participants were recruited *via* advertisements on various social networks connected to the University. Recruitment was automatically closed when five hundred persons had participated. In order to detect random answering, six verification items were included as a measure of validity throughout the survey. Two thousand six hundred and sixty-seven volunteers visited the survey site, 500 gave consent, correctly answered all the attention checks and completed the questionnaire. Participants provided written consent for data collection and analysis. Ethical approval was obtained from the local Law and Ethics Committee and the local research ethics. The research adheres to the tenets of the Declaration of Helsinki.

### Measures

#### Prodromal Questionnaire-16, Italian Version

The presence of PLEs was assessed using the Italian version of the Prodromal Questionnaire-16 (iPQ-16) (22). iPQ-16 is a 16-items self-report instrument that explores the presence/absence of 16 PLEs, including perceptual aberrations/hallucinations, unusual thought content/delusions, and two negative symptoms, and their associated psychological distress score on a four-point likert scale ranging from 0 to 48. Although the iPQ-16 was originally designed as a screening tool for individuals at Ultra-High Risk in help-seeking populations, several studies have used this instrument in non-help-seeking samples as a measure of PLEs (15, 23–25). We used the distress scale as recommended by Savill et al., (26) for non-help-seeking populations, using a cut-off of ≥11 as recommended by Pelizza et al. (27) according to the Italian field test. In this data, the average interitem correlation was 0.17 and Cronbach's α 0.85.

#### Resilience

Resilience was measured using the Resilience Scale for Adults, Italian version (28). The RSA is a 33-item scale evaluating six first order factors (Perception if Self, Planned Future, Social Competence, Structured Style, Family Cohesion and Social resources) and two second order dimensions (Personal and Contextual Resilience). RSA is a reliable (Cronbach's α from.67 to.81) and stable (test-retest, Pearson r from.73 to.80) instrument (29). RSA has a semantic differential format on a 1 to 7 likert scale with higher scores indicating stronger resilience resources.

#### Earthquake Impact

The earthquake impact was primarily explored with a single question “where were you in on the day of the earthquake?”, considering affected only those individuals that reported being in L'Aquila and surroundings on the day of the disaster. Furthermore, the entity of material damages and subjective psychological suffering due to the earthquake were assessed by two single item questions on a four-point likert scale phrased as follows: “did the earthquake cause any material loss?” and “did the earthquake cause you any psychological suffering?” with 0 = “not at all” and 4 = “extreme loss/suffering”. Due to the high number of minor shocks that preceded the main destructive one during the previous days, many citizens had already left the city when the earthquake hit L'Aquila. For this reason, material damages or psychological sufferings may have occurred independently from actually being in L'Aquila at the time of the earthquake, for example regarding material goods or significant others endangered by the earthquake, causing concern indirectly. Therefore, an impact-severity variable was computed as the sum of the scores of material damages and psychological suffering and set to zero for non-affected subjects in subsequent analysis, in order to separate the direct from the indirect traumatic experiences.

#### Potential Confounders

Potential confounders included in the survey were age, gender, family income (coded as low, mid or high), alcohol or drug abuse (coded as “abuser” if reporting “often” and “very often” use of alcohol or drugs) and family history of any mental disorder.

### Statistical Analysis

Socio-demographic characteristics, as well as mean iPQ-16 and RSA mean scores, were calculated for affected and non-affected participants. Differences between the two groups were examined using t-test or χ^2^ as appropriate.

Firstly, we compared resilience levels between affected and non-affected participants using the Mann-Whitney test for two independent samples, in order to account for non-normality of distribution of RSA values. Secondly, the unadjusted and adjusted effects of EQ, material loss, and psychological suffering on PLE were estimated using bivariate logistic regression. Finally, the unadjusted and adjusted effects of RSA on PLEs were estimated in a second wave of bivariate logistic regressions, in the whole sample and separately for subjects affected and non-affected by the EQ. In order to test any difference in the effect of Resilience on PLEs between affected and non-affected individuals, an interaction term for each RSA factor x earthquake was tested.

Due to the constraints imposed on the on-line survey, there were no missing data. However, some subjects provided invalid answers to some open questions, that were treated as missing data.

## Results

### Sample Characteristics

**Table 1** presents the characteristics of the sample. Of the 500 respondents, 6 provided invalid information on EQ impact, and were excluded from subsequent analysis. Of the remaining 494 persons, 176 (35.63%) were directly affected by the 2009 Earthquake. Mean age was 25.5 (5.84); 25 (5%) persons reported drug or alcohol abuse; 143 (28.6%), 343 (68.6%) 14 (2.8%) persons reported respectively low, middle, and high income; 76 (15.20%) reported having a 1^st^ degree relative suffering from a mental disorder. Affected and non-affected persons did not differ in terms of gender, age, drug or alcohol abuse, and income. A difference in rates of family history of mental disorder in 1^st^ degree relatives was found. In our sample, iPQ-16 distress mean score was 5.02 (s.d. 5.22); 74 (14.8%) individuals scored above cut-off.

**Table 1:** Characteristics of the sample.

Variable	Total Sample N (%)/Mean (SD)	Non-affected by EQ N (%)/Mean (SD)	Affected by EQ N(%)/Mean (SD)	Statistics (t or χ^2^)
**n**	494	318 (64.37%)	176 (35.63%)	
**Gender**				n.s.
* Female*	352 (71.26%)	230 (72.33%)	122 (69.32%)	
* Male*	142 (28.74%)	88 (27.67%)	54 (30.68%)	
**Age**	25.52 (5.85)	25.47 (5.82)	25.61 (5.92)	n.s.
**Alcohol/drug abuse**	24 (4.86%)	18 (5.66%)	6 (3.41%)	n.s.
**Income**				n.s.
* Low*	143 (28.74%)	94 (29.56%)	48 (27.27%)	
* Mid*	343 (68.42%)	218 (68.55%)	120 (68.18%)	
* High*	14 (2.83%)	6 (1.89%)	8 (4.55%)	
**Family history of mental illness**	75 (15.18%)	60 (18.87%)	15 (8.52%)	$\chi_{\text{(1)}}^{2}$ = 9.41 (p= 0.002)
**iPQ-16**				n.s.
* Mean distress score*	5.01 (5.24)	5.20 (5.39)	4.66 (4.94)	
* n≥11 distress score*	73 (14.78%)	53 (16.67%)	20 (11.36%)	
**Earthquake Impact**				n.s.
* Material damages*	0.27 (0.77)	0.07 (0.4)	0.65 (1.1)	
* Psychological suffering*	0.74 (0.97)	0.44 (0.77)	1.31 (1.04)	
* Global Impact Severity^§^*	0.51 (0.72)	0.25 (0.46)	0.98 (0.86)	

EQ, earthquake; iPQ-16, Prodromal questionnaire, Italian version; ^§^Global Impact severity is the sum of Material Loss and Psychological Suffering, in subsequent analysis it is set to 0 in non-affected persons.

### RSA in Affected and Non-Affected Persons by EQ

**Table 2** reports mean and SD for the six RSA factors and RSA total score in affected and non-affected persons by EQ, and in the total sample. The Mann-Whitney test revealed no differences in RSA factors or total score between affected and non-affected persons (all p values between 0.147 and 0.797).

**Table 2:** Resilience levels in affected and non-affected.

Variable	Total Sample	Non-affected by EQ	Affected by EQ
*Perception of Self*	4.68 (1.28)	4.66 (1.30)	4.75 (1.25)
*Planned future*	4.70 (1.51)	4.64 (1.50)	4.83 (1.55)
*Social Competence*	4.88 (1.23)	4.86 (1.25)	4.90 (1.21)
*Family cohesion*	4.92 (1.53)	4.92 (1.52)	4.91 (1.56)
*Social resources*	5.63 (1.19)	5.65 (1.15)	5.59 (1.26)
*Structured Style*	5.12 (1.22)	5.08 (1.23)	5.19 (1.21)
*RSA Total*	4.99 (0.93)	4.97 (0.93)	5.03 (0.94)

EQ, earthquake; RSA, Resilience Scale for Adults. Mann-Whitney p values ranging from 0.147 to 0.797.

### Effects of EQ on PLE

Results from logistic regression are presented in **Table 3**. No significant association was found between the 2009 earthquake and PLE [OR=0.64, 95% CI (0.37, 1.110)] nor with severity of material damages or losses [OR= 0.86, 95%CI (0.60, 1.23)] or psychological suffering [OR= 1.06, 95%CI (0.83, 1.36)]. Global impact severity was not associated with PLE [0.92 (0.76, 1.12)].

**Table 3:** Bivariate logistic regression estimates on psychotic-like experiences (PLEs) as dependent variable.

PLE	Unadjusted OR [95% CI]	Adjusted OR [95% CI]^a^
**Affected by EQ**	0.64 [0.37, 1.11]	0.82 [0.46, 1.47]
**Material Loss**	0.86 [0.60, 1.23]	0.97 [0.67, 1.40]
**Psychological Suffering**	1.06 [0.83, 1.36]	1.17 [0.90, 1.54]
**Global Impact Severity^§^**	0.92 [0.76, 1.12]	1.01 [0.83, 1.24]

EQ, Earthquake. ^a^Adjusted for age, gender, income, alcohol/drug abuse, family history of mental disorder. ^§^Global Impact Severity is the sum of Material Loss and Psychological Suffering, set to 0 in non-affected persons.

After adjustment for age, gender, income, alcohol/drug abuse, and family history of mental disorder, the results did not substantially vary.

### Effects of RSA on PLE

Results of bivariate logistic regressions of RSA factors and total score on PLE are shown in **Table 4**. Higher levels of all of the RSA factors were associated with lower prevalence PLE, with OR ranging from 0.47 [0.38, 0.59] for “Perception of Self” to 0.78 [0.64, 0.96] for “Structured Style”, and an overall OR of 0.38 [0.28, 0.51] for the RSA total score. After adjustment for age, gender, income, alcohol/drug abuse, and family history of mental disorder, the results did not vary substantially. RSA showed a stronger protective effect in affected subjects compared with non-affected individuals, with OR ranging respectively from 0.28 [0.16, 0.47] vs 0.55 [0.43, 0.70] for “Perception of Self” to 0.73 [0.5, 1.1] vs 0.81 [0.64, 1.01] for “Structured Style” factor. The RSA total score was 0.26 [0.14, 0.47] in affected subjects compared to 0.43 [0.31, 0.61] in non-affected subjects. After adjusting for age, gender, income, alcohol/drug abuse, family history of mental disorder, results did not vary substantially. Interaction terms between RSA factors and earthquake revealed a significant interaction for “Perception of Self” only [OR=0.5 (0.28, 0.90)].

**Table 4:** Bivariate logistic regression estimates of resilience on psychotic-like experiences (PLEs).

PLE	Total sample		Non-affected by EQ		Affected by EQ		Interaction term^a^
	Unadjusted OR [95% CI]	Adjusted OR [95% CI]^a^	Unadjusted OR [95% CI]	Adjusted OR [95% CI]^a^	Unadjusted OR [95% CI]	Adjusted OR [95% CI]^a^	OR [95% CI]
*Perception of Self*	0.47 [0.38, 0.59]	0.46 [0.37, 0.59]	0.55 [0.43, 0.70]	0.54 [0.42, 0,70]	0.28 [0.16, 0.47]	0.28 [0.16, 0.5]	0.5 [0.28, 0.90]
*Planned future*	0.58 [0.49, 0.69]	0.59 [0.49, 0.71]	0.62 [0.51, 0.76]	0.62 [0.50, 0.77]	0.51 [0.36, 0.70]	0.51 [0.35, 0.74]	0.81[0.55, 1.20]
*Social Competence*	0.75 [0.61, 0.91]	0.72 [0.58, 0.90]	0.78 [0.62, 0.99]	0.75 [0.58, 0.97]	0.65 [0.44, 0.96]	0.67 [0.44, 1.01]	0.83 [0.53, 1.30]
*Family cohesion*	0.71 [0.61, 0.83]	0.75 [0.63, 0.89]	0.75 [0.62, 0.90]	0.79 [0.64, 0.96]	0.61 [0.46, 0.81]	0.63 [0.46, 0.87]	0.81 [0.58, 1,10]
*Social resources*	0.61 [0.50, 0.73]	0.59 [0.48, 0.72]	0.58 [0.46, 0.75]	0.56 [0.43, 0.73]	0.61 [0.45, 0.83]	0.60 [0.42, 0.86]	1.00 [0.70, 1.50]
*Structured Style*	0.78 [0.64, 0.96]	0.84 [0.68, 1.02]	0.81 [0.64, 1.01]	0.85 [0.66, 1.10]	0.73 [0.50, 1.1]	0.77 [0.52, 1.10]	0.91 [0.58, 1.40]
*RSA Total Score*	0.38 [0.28, 0.51]	0.38 [0.28, 0.53]	0.43 [0.31, 0.61]	0.43 [0.29, 0.62]	0.26 [0.14, 0.47]	0.27 [0.14, 0.52]	0.60 [0.30, 1.20]

EQ, Earthquake. Adjusted for age, gender, income, alcohol/drug abuse, family history of mental disorder. ^a^Interaction term, EQ x RSA factors. RSA, Resilience Scale for Adults.

## Discussion

### Summary of Findings

Long term effects of natural disasters, such as the 2009 L'Aquila earthquake, on PLEs are mostly unknown. We unexpectedly found lower levels of PLEs in affected adolescents compared to non-affected ones right after the EQ (13). In this study, we explored the effects on PLEs 10 years after an earthquake in a University student sample. No effects of EQ, material loss or psychological suffering on the rates of PLE were found. Resilience was confirmed as a strong protective factor for PLEs, with higher scores on all RSA factors being associated with lower rates of PLEs, irrespective of a natural disaster. Indeed, resilience levels did not differ between affected and non-affected persons. However, the protective effect of resilience as a total score, and “perception of self” factor in particular, was substantially stronger in subjects affected by the earthquake.

### Comparison With Previous Literature

To the best of our knowledge, this is one of the few reports investigating the very long-term effects of a natural disaster on PLE. Overall, only a limited and conflicting evidence on the impact of natural disasters on PLEs exists (6). Ayub and colleagues (11) reported increased rates of psychotic symptoms after the 2005 Kashmir earthquake, although the absence of a control group didn't allow them to estimate the effects of the EQ on PLE. In a national survey in Sri Lanka (12), an association between PLE and being affected by a tsunami, occurred 10 years before, was found. However, such association did not hold after controlling for conflict-related trauma. Moreover, no effect on PLEs was found in a 20 year follow-up study on PLEs in individuals affected by bushfires in Australia (30). Data from the World Mental Health survey confirmed a weak effect of natural disaster of unspecified type on PLEs, that did not hold after controlling for confounders (2).

In our sample, the prevalence of PLEs is considerably higher than the lifetime prevalence of around 5% reported in the literature (31). This could be due to the use of a self-report instrument that could have overestimated PLE prevalence compared to semi-structured interviews. Estimates of the prevalence of PLEs in population-based samples vary considerably, mostly depending on the type of instrument used (3, 31–33). The prevalence in our sample is similar to a 19.1% found in another study using the Prodromal Questionnaire in a community sample (9). In this study, however, the number of endorsed items, rather than the distress score, was taken into account, contrary to what suggested by Savill and colleagues (26).

### Meaning

Our findings represent the first report on PLEs at a such long time after a natural disaster. To the best of our knowledge, the interactions between resilience and PLEs in response to a natural disaster have not been addressed so far. Moreover, the generally accepted theory is that traumatic events, independently of their type, negatively affect PLE (6). Our current findings, if confirmed by larger and more rigorous epidemiological studies, could challenge this assumption, suggesting to reconsider the effect of natural disasters on PLEs rates in the light of individual or contextual resilience factors (34, 35).

Non-deterioration or even improvement in psychotic symptoms soon after being affected by a man-made disaster, including terror attacks, has been already proposed (36). Among the potential protective factors, resilience is of pivotal importance in contrasting the effects of traumatic events and in mitigating the impact of psychological symptoms (18, 37). Consistently, resilience levels did not differ between affected and non-affected individuals in our sample. The mean age in our sample is 25, which means that participants were, on the average, in their adolescence when affected by the earthquake. This is a critical aspect, because stressful life events have their major impact when occurring during adolescence. However, assuming that risk factors play their strongest disruptive effect during adolescence, it is reasonable to state that also protective factors could exert their maximum protection during the same period. Resilience has already been involved in mitigating the onset of PLEs (16). Our findings suggest that the 2009 EQ affected a resilient population that may have bounced back during the immediate aftermaths of the EQ, and that may have fully recovered, in terms of PLE rates, 10 years after. If the EQ produced immediate psychological symptoms, resilience could have promoted a quick subjective process of personal recovery in heavily traumatized individuals (38).

The exact protective mechanisms of resilience on PLEs remain elusive. Our finding of a stronger protective effect in affected *vs* non-affected subjects suggests that resilience could be a personal resource particularly important following a natural disaster. It could be carefully speculated that, if on the one hand, resilience is particularly important in long-term protection from natural disasters, other factors could play a protective role in persons not involved in traumatic experiences.

In our results, the resilience factor showing the strongest protective effect was “perception of self”, a factor ﻿concerning the confidence in one's own abilities, self-confidence, self-efficacy, positive outlook. This is coherent with evidence low self-esteem mediating the effects of childhood adversities on PLEs (5). It is noteworthy that this factor in particular show very different OR between affected and non-affected subjects.

Further studies are needed to explore the pathways through different resilience factors.

### Limitations

The present study has several limitations. Firstly, it has a cross-sectional design, hence reports on earthquake impact, especially on psychological suffering, may have been affected by recall bias. However, more objective measures such as material damages should be less sensitive to recall bias. Secondly, a considerable number of people had left the city of L'Aquila, possibly due to long-lasting psychological problems associated with constant re-exposure to traumatic reminders of the earthquake during the last 10 years (39) [based on the authors' personal experience. See also (40)]. This could have introduced a selection bias with the underrepresentation of heavily traumatized subjects. Thirdly, our study was based on voluntary on-line recruitment, that could have introduced a self-selection bias. Finally, our study lacks a measure of post-traumatic symptoms that could have provided further in-depth analysis of the relations between this particular traumatic event and PLEs.

The major limitation of this study is that it lacks a baseline assessment of resilience prior to the earthquake, or any comparable measure of resilience in the general population or in another comparable convenience sample. Moreover, severity of psychological sufferings was not measured using a standardized psychometric instrument.

## Conclusions

This is the first study reporting the very-long term impact of a natural disaster in PLEs in a university student sample of young adults. No direct effect of the earthquake on PLEs was detected after 10 years after disaster. Resilience could have played a key role in normalizing the psychopathological effect of such a traumatic experience. Further studies are required in order to better characterize individual and contextual determinants of resilience in the face of a traumatic event. Moreover, the evidence of a lack of long-term effect of a natural disaster on PLEs needs to be replicated in different populations.

## Data Availability Statement

The datasets generated for this study are available on request to the corresponding author.

## Ethics Statement

The studies involving human participants were reviewed and approved by the internal review board at University of L'Aquila. The patients/participants provided their written informed consent to participate in this study.

## Author Contributions

Conceptualization, RR, AC, PS, FP, and AR. Methodology: R.R and AC. Software: RR and AC. Formal analysis: RR. Data curation: AC and RR. Writing—original draft preparation: RR. Writing—review and editing: RR, DT, AR, PS, EG, VS, FP and GL.

## Conflict of Interest

The authors declare that the research was conducted in the absence of any commercial or financial relationships that could be construed as a potential conflict of interest.

## References

[1] Alexander DE Ten years after the 6 April 2009 L"Aquila earthquake: some reflections. (2019).

[2] McGrath JJ Saha S Lim CCW Aguilar-Gaxiola S Alonso J Andrade LH Trauma and psychotic experiences: Transnational data from the World Mental Health survey. Br J Psychiatry (2017) 211(6):373–80. 10.1192/bjp.bp.117.205955 PMC5709675 29097400

[3] Linscott RJ Van Os J An updated and conservative systematic review and meta-analysis of epidemiological evidence on psychotic experiences in children and adults: on the pathway from proneness to persistence to dimensional expression across mental disorders. Psychol Med (2013) 43(6):1133–49. 10.1017/S0033291712001626 22850401

[4] Rossi R Zammit S Button KS Munafò MR Lewis G David AS Psychotic experiences and working memory: a population-based study using signal-detection analysis. PloS One (2016) 11(4):e0153148. 10.1371/journal.pone.0153148 27120349 PMC4847914

[5] Fisher HL Schreier A Zammit S Maughan B Munafò MR Lewis G Pathways between childhood victimization and psychosis-like symptoms in the ALSPAC birth cohort. Schizophr Bull (2013) 39(5):1045–55. 10.1093/schbul/sbs088 PMC3756772 22941743

[6] Gibson LE Alloy LB Ellman LM Trauma and the psychosis spectrum: a review of symptom specificity and explanatory mechanisms. Clin Psychol Rev (2016) 49:92–105. 10.1016/j.cpr.2016.08.003 27632064 PMC5157832

[7] Stratta P Sanità P Bonanni RL de Cataldo S Angelucci A Rossi R Clinical correlates of plasma brain-derived neurotrophic factor in post-traumatic stress disorder spectrum after a natural disaster. Psychiatry Res (2016) 244:165–70. 10.1016/j.psychres.2016.07.019 27479108

[8] Bhavsar V Boydell J McGuire P Harris V Hotopf M Hatch SL Childhood abuse and psychotic experiences - evidence for mediation by adulthood adverse life events. Epidemiol Psychiatr Sci (2019) 28(3):300–9. 10.1017/S2045796017000518 PMC5784808 28988558

[9] Mongan D Shannon C Hanna D Boyd A Mulholland C The association between specific types of childhood adversity and attenuated psychotic symptoms in a community sample. Early Interv Psychiatry (2019) 13(2):281–9. 10.1111/eip.12478 28941143

[10] Farooqui M Quadri SA Suriya SS Khan MA Ovais M Sohail Z Posttraumatic stress disorder: a serious post-earthquake complication. Trends Psychiatry Psychother (2017) 39(2):135–43. 10.1590/2237-6089-2016-0029 28700042

[11] Ayub M Saeed K Kingdon D Naeem F Rate and predictors of psychotic symptoms after Kashmir earthquake. Eur Arch Psychiatry Clin Neurosci (2015) 265(6):471–81. 10.1007/s00406-014-0561-1 25421792

[12] Keraite A Sumathipala A Siriwardhana C Morgan C Reininghaus U Exposure to conflict and disaster: a national survey on the prevalence of psychotic experiences in Sri Lanka. Schizophr Res (2016) 171(1–3):79–85. 10.1016/j.schres.2016.01.026 26817400

[13] Rossi A Di Tommaso S Stratta P Riccardi I Daneluzzo E How much stress is needed to increase vulnerability to psychosis? A community assessment of psychic experiences (CAPE) evaluation 10 months after an earthquake in L'Aquila (Italy). Eur Arch Psychiatry Clin Neurosci (2012) 262(3):265–8. 10.1007/s00406-011-0258-7 21932082

[14] Rossi A Carmassi C Daneluzzo E Stratta P Riccardi I Marino A Community Assessment of Psychic Experiences (CAPE) and Trauma and Loss Spectrum (TALS) 12 months after an earthquake in Italy. J Psychopathol (2013) 19(1):68–72.

[15] Mętel D Arciszewska A Daren A Pionke R Cechnicki A Frydecka D Mediating role of cognitive biases, resilience and depressive symptoms in the relationship between childhood trauma and psychotic-like experiences in young adults. Early Interv Psychiatry (2019), 14:87–96. 10.1111/eip.12829 31099186

[16] Crush E Arseneault L Moffitt TE Danese A Caspi A Jaffee SR Protective factors for psychotic experiences amongst adolescents exposed to multiple forms of victimization. J Psychiatr Res (2018) 104:32–8. 10.1016/j.jpsychires.2018.06.011 PMC6109202 29929082

[17] Galderisi S Rossi A Rocca P Bertolino A Mucci A Bucci P The influence of illness-related variables, personal resources and context-related factors on real-life functioning of people with schizophrenia. World Psychiatry (2014) 13(3):275–87. 10.1002/wps.20167 PMC4219069 25273301

[18] Rossi A Galderisi S Rocca P Bertolino A Rucci P Gibertoni D Personal resources and depression in schizophrenia: the role of self-esteem, resilience and internalized stigma. Psychiatry Res (2017) 256:359–64. 10.1016/j.psychres.2017.06.079 28686933

[19] Galderisi S Rucci P Kirkpatrick B Mucci A Gibertoni D Rocca P Interplay among psychopathologic variables, personal resources, context-related factors, and real-life functioning in individuals with schizophrenia a network analysis. JAMA Psychiatry (2018) 75(4):396–404. 10.1001/jamapsychiatry.2017.4607 29450447 PMC5875306

[20] Stratta P de Cataldo S Bonanni R Valenti M Masedu F Rossi A Mental health in L'Aquila after the earthquake. Ann Ist Super Sanita (2012) 48(2):132–7. 10.4415/ANN_12_02_05 22751555

[21] Stratta P Capanna C Patriarca S de Cataldo S Bonanni RL Riccardi I Resilience in adolescence: gender differences two years after the earthquake of L'Aquila. Pers Individ Dif (2013) 54(3):327–31. 10.1016/j.paid.2012.09.016

[22] Azzali S Pelizza L Paterlini F Garlassi S Scazza I Chiri LR Reliability of the Italian version of the 16-item Prodromal Questionnaire (iPQ-16) for psychosis risk screening in a young help-seeking community sample. J Psychopathol (2018) 24(1):16–23.

[23] Gawęda Ł Göritz AS Moritz S Mediating role of aberrant salience and self-disturbances for the relationship between childhood trauma and psychotic-like experiences in the general population. Schizophr Res (2019) 206:149–56. 10.1016/j.schres.2018.11.034 30545759

[24] Sengutta M Gawęda Ł Moritz S Karow A The mediating role of borderline personality features in the relationship between childhood trauma and psychotic-like experiences in a sample of help-seeking non-psychotic adolescents and young adults. Eur Psychiatry (2019) 56:84–90. 10.1016/j.eurpsy.2018.11.009 30599337

[25] Gawęda Ł Pionke R Krężołek M Frydecka D Nelson B Cechnicki A The interplay between childhood trauma, cognitive biases, psychotic-like experiences and depression and their additive impact on predicting lifetime suicidal behavior in young adults. Psychol Med (2019) 10:1–9. 10.1017/S0033291718004026 30626466

[26] Savill M D'Ambrosio J Cannon TD Loewy RL Psychosis risk screening in different populations using the Prodromal Questionnaire: a systematic review. Early Interv Psychiatry (2018) 12(1):3–14. 10.1111/eip.12446 PMC5812357 28782283

[27] Pelizza L Azzali S Paterlini F Garlassi S Scazza I Pupo S The Italian version of the 16-item prodromal questionnaire (iPQ-16): Field-test and psychometric features. Schizophr Res (2018) 199:353–60. 10.1016/j.schres.2018.03.023 29571752

[28] Capanna C Stratta P Hjemdal O Collazzoni A Rossi A The Italian validation study of the Resilience Scale for Adults (RSA). Appl Psychol Bull (2015) 272(63):16–24.

[29] Hjemdal O Friborg O Stiles TC Rosenvinge JH Martinussen M Resilience predicting psychiatric symptoms: a prospective study of protective factors and their role in adjustment to stressful life events. Clin Psychol Psychother (2006) 13(3):194–201. 10.1002/cpp.488

[30] Galletly C Van Hooff M McFarlane A Psychotic symptoms in young adults exposed to childhood trauma—A 20year follow-up study. Schizophr Res (2011). 127:76–82. 10.1016/j.schres.2010.12.010 21256719

[31] McGrath JJ Saha S Al-Hamzawi A Alonso J Bromet EJ Bruffaerts R Psychotic experiences in the general population. JAMA Psychiatry (2015) 72(7):697. 10.1001/jamapsychiatry.2015.0575 26018466 PMC5120396

[32] Kelleher I Cannon M Psychotic-like experiences in the general population: Characterizing a high-risk group for psychosis. Psychol Med (2011). 10.1017/S0033291710001005 20624328

[33] Niarchou M Zammit S Lewis G The Avon Longitudinal Study of Parents and Children (ALSPAC) birth cohort as a resource for studying psychopathology in childhood and adolescence: a summary of findings for depression and psychosis. Soc Psychiatry Psychiatr Epidemiol (2015) 50(7):1017–27. 10.1007/s00127-015-1072-8 26002411

[34] Southwick SM Bonanno GA Masten AS Panter-Brick C Yehuda R Resilience definitions, theory, and challenges: Interdisciplinary perspectives. Eur J Psychotraumatol (2014) 5(1):25338. 10.3402/ejpt.v5.25338 PMC4185134 25317257

[35] Bonanno GA Brewin CR Kaniasty K La Greca AM Weighing the costs of disaster: consequences, risks, and resilience in individuals, families, and communities. Psychol Sci Public Interes Suppl (2010) 11(1):1–49. 10.1177/1529100610387086 26168411

[36] Katz CL Pellegrino L Pandya A Ng A DeLisi LE Research on psychiatric outcomes and interventions subsequent to disasters: a review of the literature. Psychiatry Res (2002). 41:1–6. 10.1017/S0033291710001005 12127471

[37] Rossetti MC Tosone A Stratta P Collazzoni A Santarelli V Guadagni E Different roles of resilience in depressive patients with history of suicide attempt and no history of suicide attempt. Rev Bras Psiquiatr (2017) 39(3):216–9. 10.1590/1516-4446-2016-2045 PMC7111383 28538755

[38] Rossi A Amore M Galderisi S Rocca P Bertolino A Aguglia E The complex relationship between self-reported ‘personal recovery' and clinical recovery in schizophrenia. Schizophr Res (2018) 192:108–12. 10.1016/j.schres.2017.04.040 28495492

[39] Pagani M Di Lorenzo G Monaco L Daverio A Giannoudas I La Porta P Neurobiological response to EMDR therapy in clients with different psychological traumas. Front Psychol (2015) 6(OCT):1614. 10.3389/fpsyg.2015.01614 26579006 PMC4621396

[40] National demographic balance [Internet]. Available at: https://www.istat.it/en/archivio/232013 (Accessed August 29, 2019).

